# Supplementary material for: Quantitative measurement of the histological features of alpha-1 antitrypsin deficiency-associated liver disease in biopsy specimens
Source: PLoS One. 2021 Aug 16;16(8):e0256117. doi: 10.1371/journal.pone.0256117 (PMC8366994; doi:10.1371/journal.pone.0256117)
Supplement: S1 Text — (DOCX) [file pone.0256117.s001.docx]

**Supporting Methods**

***Quantification of PAS-D Inclusions***

As a simple method to quantify PAS-D material we tested a thresholded positive pixel count to detect PAS-D inclusions (**Fig A)**. However, this overestimates AAT accumulation and does not individually quantify PAS-D inclusions.

The quantitative method we created takes advantage of color de-mixing, thresholding and particle analysis to provide a relative quantification and size measurement of PAS-D inclusions. We measured globules using background subtracted whole-slide images. Color deconvolution was used to separate PAS, hematoxylin and lipofuscin material in slides based on RGB values calculated using the Colour-Deconvolution plugin in FIJI.(1) (2) Our algorithm creates a selection of the total tissue area by 8-bit thresholding allowing for normalization to the amount of tissue that is present. The PAS layer is auto-thresholded to 80% of maximum using the “DEFAULT” imageJ method. After thresholding, the image is converted to a binary mask, holes in pixel intensity are filled with a binary operation and then the resultant image is eroded followed by a dilation to remove background intensities. Finally, particle analysis is applied to the binary mask to detect inclusions that are between delimited size and circularity constants in the normalized biopsy area (**Fig. S1B-C)**. We have provided a macro-operation that runs these scripts on all images in a directory and outputs a summary spreadsheet with the count, area and percent area detected and individual sizes for each inclusion and a merged image with every detected inclusion overlaid with the original image. Tuning of the algorithm was accomplished by first setting the thresholding to separate higher intensity PAS-D staining, then tuning size and circularity in particle analysis to arrive at results consistent with human observers example settings are shown in (**Table B).** All measurements in this study were developed on a modestly upgraded computer workstation. Processing whole slide images with these methods requires substantial random access memory, we suggest a workstation with a minimum of 32GB RAM for smooth operation of these macros on whole slide images.

| **Table A: Antibodies and IHC Parameters** | | | | | |
| --- | --- | --- | --- | --- | --- |
|  | | | | | |
| **Primary**  **Antibody** | **Vendor** | **Catalog#** | **Antigen Retrieval** | **Dilution** | **Secondary** |
| 2C1 AAT | Hycult | HM2289 | Citra | 1:1000 | Mouse HRP |
|  |  |  |  |  |  |
| AAT | Fitzgerald | RDI-A1ATRYPabr | Trypsin | 1:600 | Rabbit HRP |
|  |  |  |  |  |  |
| LC3B | Novusbio | NB100-2220 | Citra | 1:500 | Rabbit HRP |
|  |  |  |  |  |  |
| Annexin V | Abcam | Ab54775 | Citra | 1:300 | Mouse HRP |
|  |  |  |  |  |  |

**Table A: Antibodies used for Immunohistochemistry**

Specific antibodies dilutions and antigen retrieval methods used for immunohistochemistry.

| **Table B: Algorithm Tuning** | | | | | |
| --- | --- | --- | --- | --- | --- |
| **Parameters** | **Algorithm 1** | **Algorithm 2** | **Algorithm 3** | **Algorithm 4** | **Algorithm 5** |
| **Threshold** | 0-max/1.25 | 0-max/1.25 | 0-max/1.25 | 0-max/1.25 | 0-max/1.25 |
| **Size (um^2^)** | 1-500 um | 1-500 um | 5-500 um | 5-500 um | 5-500 um |
| **Circularity %** | 50% | 60% | 70% | 60% | 50% |

**Table B, Algorithm Tuning,** Selected tuning parameters used to developing quantitative inclusion analysis

***Immunohistochemistry***

Slides were de-paraffinized with xylene and re-hydrated through decreasing concentrations of ethanol to water, including an intermediary step to quench endogenous peroxidase activity (3% hydrogen peroxide in methanol). Slides were transferred to 1X TBS-T (Tris-buffered saline-Tween). For heat-induced antigen retrieval, sections were heated in a steamer while submerged in Citra buffer (Biogenex, Fremont, CA) for 30 minutes. Slides were subsequently rinsed in 1XTBS-T and incubated with a universal protein blocker Sniper (Biocare Medical, Walnut Creek, CA), for 15 minutes at room temperature. Slides were rinsed in 1XTBT-T and co-incubated in primary antibody or isotype control for 1 hour at RT. Slides were rinsed in 1XTBS-T followed by application of conjugated secondary antibody: Mach 2 goat anti-Rabbit or Mouse (choose one) horse radish peroxidase-conjugated (Biocare Medical, Walnut Creek, CA) for 30 minutes at room temperature. Detection was achieved by incubating slides in 3’3’ diaminobenzidine (Biocare Medical, Walnut Creek, CA) for 1-2 minutes at RT. Slides were counterstained with hematoxylin (Vector Laboratories Inc., Burlingame, CA) for 30 seconds and mounted with Cytoseal XYL (Richard-Allen Scientific, Kalamazoo, MI).

***Quantification of Immunohistochemical Staining***

Biopsy specimens were stained with total AAT and polymer specific (2C1) antibodies and compared to slides prepared with non-specific IgG primary antibody.

After scanning, the image data was background subtracted and color de-mixed, prior to thresholding via “DEFAULT” method positive pixels were thresholded between 0- and two thirds of the maximum calculated pixel intensity. Proportional area of staining was calculated as the percent DAB positive pixels to Total Biopsy Area. Analysis of Total-IHC AAT and 2C1 IHC AAT was carried out in batch using the ImageJ-Macro provided. Polymer specific immunohistochemistry was performed using 2C1 Antibody (Hycult Biotech) Total-AAT IHC was performed at the UF Health Pathology Laboratories.

***PAS-D Macro***

This code is provided in image j macro language.

(//) Indicates unread lines that are intended to describe functions.

To use these methods, copy and paste the following code into a new macro in ImageJ (FIJI)

**Code:**

//Set the directory for input and output folders and files

run("Bio-Formats Macro Extensions");

dir1 = getDirectory("Choose Source Directory ");

dir2 = getDirectory("Choose Destination Directory ");

list = getFileList(dir1);

setBatchMode(true);

for (i=0; i<list.length; i++) {

showProgress(i+1, list.length);

Ext.openImagePlus(dir1+list[i]);

imgName=getTitle();

//First set the measurements to work in um based on scale of the image detector

run("Set Measurements...", "area limit display redirect=None decimal=3");

run("Set Scale...", "distance=400 known=200 pixel=1 unit=uM global");

run("RGB Color");

selectWindow(imgName);

close();

imgName=getTitle();

//Step1: subtract background, color separate color spectra

run("Subtract Background...", "rolling=50 light");

run("Colour Deconvolution", "vectors=[User values] [r1]=0.64 [g1]=0.72 [b1]=0.27 [r2]=0.064 [g2]=0.99 [b2]=0.095 [r3]=0.27 [g3]=0.80 [b3]=0.53");

//close windows we don't need

selectWindow("Colour Deconvolution");

run("Close");

selectWindow(imgName +"-(Colour_3)");

title = getTitle();

run("Close");

//duplicate image for merging with markup at the end

selectWindow(imgName);

title = getTitle();

run("Duplicate...", " ");

//Select the total tissue area in the sample

selectWindow(imgName);

title = getTitle();

run("8-bit");

setAutoThreshold("Default");

setThreshold(0, 250);

run("Create Selection");

//measure the total size of the tissue, close this markup

run("Measure");

close();

//Select the PAS-D material, remove background

selectWindow(imgName +"-(Colour_2)");

title = getTitle();

run("Subtract Background...", "rolling=100 light");

//Autothreshold pixels to detect globules, this can be set manually or adjusted based on the samples

setAutoThreshold("Default");

getThreshold(min, max);

setThreshold(0, max/1.25);

setOption("BlackBackground", false);

//create binary image and fill holes in circular intensities, followed by cleanup of single pixel intensities

run("Convert to Mask");

run("Fill Holes");

run("Open");

//select the entire tissue area as measured above

run("Restore Selection");

//Analyze particles and create markups

run("Analyze Particles...", "size=5-500 circularity=0.6-1.00 show=Outlines display include summarize");

//stack images and make a merged image between measured globules and original image

title = getTitle();

selectWindow(imgName +"-(Colour_2)");

run("Close");

run("Images to Stack", "name=Stack title=[] use");

selectWindow("Stack");

run("Z Project...", "projection=[Average Intensity]");

saveAs("Tiff", dir2 + imgName + "merge.tif");

//close windows, save individual image results

close();

selectWindow("Stack");

close();

saveAs("Results", dir2 + imgName +"globules.xls");

//cleanup results before processing the next image, clear working memory

run("Clear Results");

run("Close All");

call("java.lang.System.gc");

//go to next image

}

//after all images have been processed, save summary sheet

selectWindow("Summary")

saveAs("Results", dir2 + "Summary.xls");

**//END OF CODE**

***IHC Macro Code***

This code is provided in ImageJ macro language.

(//) Indicates unread lines that are intended to describe functions.

To use these methods copy and paste the following code into a new macro in imageJ (FIJI)

**Code:**

run("Bio-Formats Macro Extensions");

dir1 = getDirectory("Choose Source Directory ");

dir2 = getDirectory("Choose Destination Directory ");

list = getFileList(dir1);

setBatchMode(true);

for (i=0; i<list.length; i++) {

showProgress(i+1, list.length);

Ext.openImagePlus(dir1+list[i]);

imgName=getTitle();

//set measurement properties

run("Set Measurements...", "area integrated area_fraction limit display redirect=None decimal=3");

selectWindow(imgName);

//prepare image for measurements

run("RGB Color");

call("java.lang.System.gc");

selectWindow(imgName +" (RGB)");

run("Subtract Background...", "rolling=20 light");

run("Colour Deconvolution", "vectors=[H DAB]");

selectWindow(imgName +" (RGB)-(Colour_1)");

title = getTitle();

run("Close");

call("java.lang.System.gc");

selectWindow(imgName +" (RGB)-(Colour_3)");

title = getTitle();

run("Close");

call("java.lang.System.gc");

//Select the specimen from within the total area of the image

selectWindow(imgName +" (RGB)");

run("8-bit");

setThreshold(0, 250);

run("Create Selection");

close();

call("java.lang.System.gc");

//Threshold the stained area within the specimen and measure

selectWindow(imgName +" (RGB)-(Colour_2)");

title = getTitle();

setAutoThreshold("Default");

getThreshold(min, max);

setThreshold(0, max/1.5);

run("Restore Selection");

run("Measure");

//Save representative images and close windows

saveAs("Tiff", dir2 + imgName + " (RGB)-(Colour_2).tif");

run("Select None");

setAutoThreshold("Default");

getThreshold(min, max);

setThreshold(0, max/1.5);

run("Create Selection");

selectWindow(imgName);

run("RGB Color");

selectWindow(imgName);

close();

selectWindow(imgName +" (RGB)");

run("Restore Selection");

setForegroundColor(255, 0, 0);

fill();

saveAs("Tiff", dir2 + imgName + "merge.tif");

close();

selectWindow("Colour Deconvolution");

close();

call("java.lang.System.gc");

selectWindow(imgName + " (RGB)-(Colour_2).tif");

close();

call("java.lang.System.gc");

//Save Results

selectWindow("Results");

saveAs("Results", dir2 + "AAT_IHC_area.xls");

run("Close All");

call("java.lang.System.gc");

}

**//END OF CODE**

**Computer Equipment Specifications:**

*Operating system:*

Microsoft Windows 7 Enterprise 64-bit

*MicroProcessor:*

Intel Xenon CPU E5-1650 V2 3.49 ghz

*RAM:*

96 gb 1600 hz

**References Supplementary Material**

1. Schindelin J, Arganda-Carreras I, Frise E, Kaynig V, Longair M, Pietzsch T, Preibisch S, et al. Fiji: an open-source platform for biological-image analysis. Nat Methods 2012;9:676-682.

2. Ruifrok AC, Johnston DA. Quantification of histochemical staining by color deconvolution. Anal Quant Cytol Histol 2001;23:291-299.
