# Supplementary figures and images for: Quantitative measurement of the histological features of alpha-1 antitrypsin deficiency-associated liver disease in biopsy specimens
Source: PLoS One. 2021 Aug 16;16(8):e0256117. doi: 10.1371/journal.pone.0256117 (PMC8366994; doi:10.1371/journal.pone.0256117)

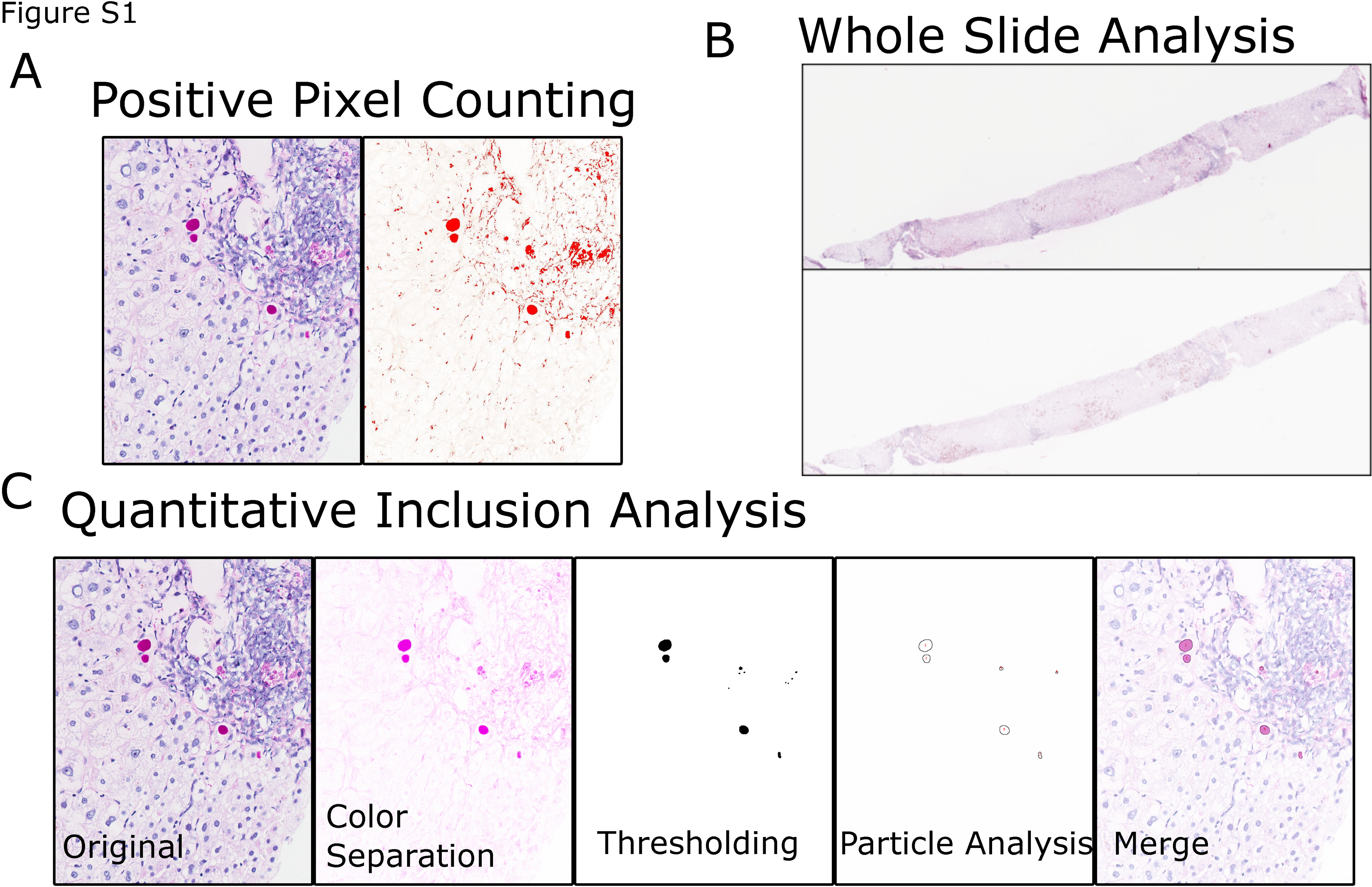

Supplement: S1 Fig — (A) Positive pixel counting in PAS-D slides over-represents accumulation in humans (B) Representation of whole slide processing low resolution (C) Representation of steps involved in inclusion analysis. (TIF) [file pone.0256117.s002.tif]

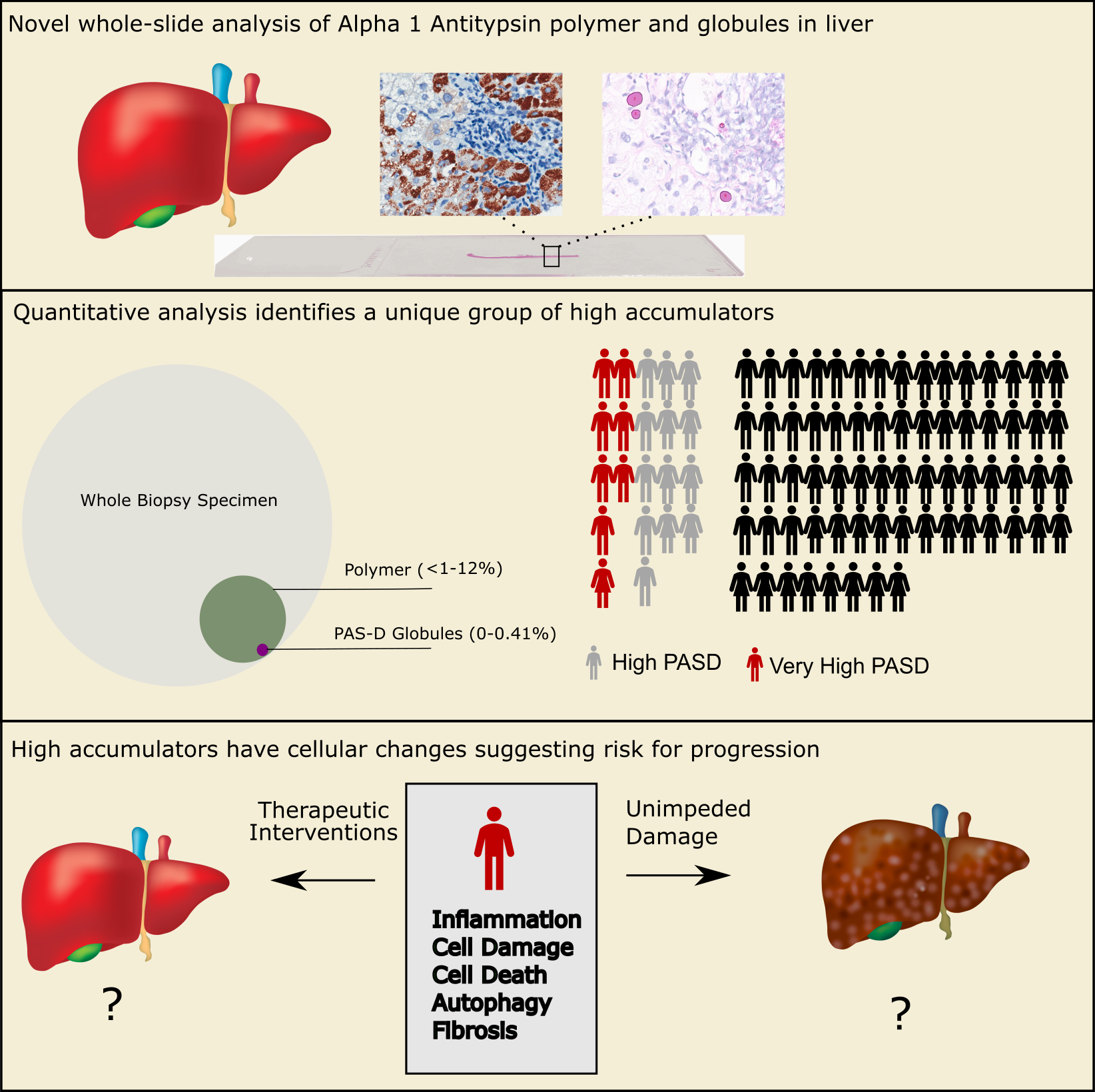

Supplement: S1 Graphical abstract — Top panel, whole slide imaging of PAS-D and polymer specific IHC, Middle panel, median PAS-D, Polymer and Total IHC shows the different fractions and quantitative analysis identifies specific individuals who has high and very high AAT accumulation. Bottom panel, High accumulators identified by inclusion analysis may be at higher risk of progressive disease and theoretically may benefit from future interventions. (TIF) [file pone.0256117.s004.tif]
