# Supplementary material for: Quantitative measurement of the histological features of alpha-1 antitrypsin deficiency-associated liver disease in biopsy specimens
Source: PLoS One. 2021 Aug 16;16(8):e0256117. doi: 10.1371/journal.pone.0256117 (PMC8366994; doi:10.1371/journal.pone.0256117)
Supplement: Attachment — Submitted filename: Response to Reviewers.pdf [file pone.0256117.s005.pdf]

UF UNIVERSITY of FLORIDA

Department of Medicine
Division of Pulmonary Critical Care and Sleep Medicine

Alpha 1 Research Program
PO Box 100225
1600 SW Archer Rd, M-333
Gainesville, FL 32611-0225

Dear Dr. Strnad and Review Committee,

Thank you for reviewing our work and offering us the opportunity to incorporate the comments and revisions requested by the review committee. Our paper entitled *Quantitative measurement of the histological features of alpha-1 antitrypsin deficiency-associated liver disease in biopsy specimens* uses whole slide imaging to study baseline histologic data from a large longitudinal cohort of people who have PiZZ alpha-1 antitrypsin deficiency. We demonstrate and provide new tools which can be used to estimate severity of alpha 1 antitrypsin accumulation in standard pathology specimens. We have addressed each reviewers major and minor revisions as below. We have also made editorial revisions as noted.

Editorial Revisions

1) We have addressed the style requirements and file naming for PLOS ONE
2) We have clarified this in human subjects section, see line 141
3) We have included an additional participant recruitment section which details this information, Table 1 provides basic demographic information for the population and more extensive details have been previously published in our original descriptive work [8]
4) Figure 6 (graphical summary) has been uploaded
5) We have updated the supplementary material as requested
6) With respect to Dr. Collinsworth's affiliation with a commercial histopathology group:

Financial Interests Statement:

Dr. Collinsworth is currently affiliated with Advanced Pathology Solutions however she was faculty at University of Florida during the work included in this project. Advanced Pathology Solutions was not involved in the funding, design or execution of the work presented and did not provide relevant salary or other support to Dr. Collinsworth or any members of the research team during her work on this project.

Competing Interests Statement:

Dr. Amy Collinsworth is currently employed at Advanced Pathology Solutions. However, her work on this project was completed while faculty at the University of Florida. Advanced Pathology Solutions was not a part of the design or funding of this project and she has not been involved in work on this project since her transition to her new commercial affiliation. Dr. Collinsworth's current affiliation

does not alter our adherence to PLOS ONE policies on sharing data and materials and Advanced Pathology Solutions has had no research interest in our work.

Reviewer #1

Reviewer #1 had specific major concerns related to the generalizability of our methods to wider use and was interested in longitudinal and comparisons of PAS-D accumulation with other known metabolic diseases, sex and aging.

1. We certainly agree that histological analysis should not be overestimated. One advantage of this method is that it was designed around a standard stain (PAS-D) which is used across many institutions and is standard practice for evaluating liver biopsies of most people who have had a liver biopsy should have a PAS-D slide archived. Even the path specimens in our cohort were not strictly without differences in hue and stain intensity which is why background subtraction tools, color deconvolution and binary operations are used.

2. We have some data available, including yearly non-invasive measurements which suggest association between worsening transaminases and liver stiffness measurements and AST to platelet ratio index which we plan to analyze together with other clinical markers together as part of forthcoming publications.

3. This is a very appropriate question, the answer is yes, but we have already published these relationships in our original paper discussing this same cohort and with our qualitative scores.

Minor comments:
- We made these changes as requested.

Reviewer #2

Reviewer #2 had several major revisions and comments. One important feature of these comments which needs to be clarified is the nature of our measurements. There are several questions discussing the data in terms of standard methods using high-powered fields. It is very important to note that we are providing a method to estimate globule area in whole slide images. This is extensively discussed in our methods and supplementary material and is essential to interpretation of our data. We have addressed this and additional concerns including requests for additional statistical analysis and details as below.

1. The data is presented as % area because we analyzed whole slides and globules have both size and number that contribute to how much of a biopsy is occupied.

2. We have added additional data about the size and number of globules including their confidence intervals which helps to quantify these differences. Additionally, our histologic polymer analysis shows very clearly that only a portion of any given individual’s liver is occupied by polymer and

differences in hepatocellular accumulation of AAT are discussed with relation to hepatocyte degeneration and autophagy in results and discussion of this paper. Additionally, our original publication on the clinical characteristics also describes the zonal distribution of globules and has already been published.

3. With respect to the relationship between globules and polymer, the original statement is correct and is supported by our data showing polymer staining occupies a larger proportion of the biopsies than PAS-D aggregates alone. PAS-D aggregates are large aggregates of polymer, but not all polymer is within a PAS-D aggregate. Total AAT antibodies stain both polymeric and non-polymeric AAT, and total AAT staining did not have a relationship with other aspects of liver pathology. See also Fig 6 which we added in our revised manuscript.

4. We added additional statements in the methods and supplement to help clarify this point. The method is based on a whole slide analysis, which means the whole slide is scanned initially, which includes background white space. Our analysis measures the area of the biopsy based on total pixels that are positive after background subtraction. The measurements of globules within this area are then normalized the total area of each biopsy so that the size of the biopsy ultimately does not impact the quantification by this method.

5. This is whole slide imaging, so every field on a biopsy is counted. This is a seminal feature of our work in that the quantification of PAS-D is not biased by the number or location where fields are chosen.

6. The counts are whole slide analysis so this data represents all the annexin foci in the entire biopsy and divided by the size of tissue in mm^2 so it is not a representation of a number of fields of view.

7. Polymer IHC was examined on the same population as PAS-D. The individual point values are shown in figure 2. Four samples couldn't be analyzed because of tissue constraints, and total staining has high background and artifacts, so 7 samples couldn't be used for this analysis.

8. We did do these analyses as well. Log transformed data reduced the skew and created a normal distribution for running ANOVA but this did not change the overall conclusions of the data.

9. We developed the algorithms using lins statistic comparing the observers to five putative algorithms and chose the best from here. I have added this information to the methods and results.

10. The explanted specimens used are archived slides some of which come from early days of the DNA and tissue bank. Very few of these are available from explanted cirrhosis tissue and they patients are not well characterized. For our purposes we examined them to measure PAS-D to provide the upper extremes of disease because our cohort of well characterized biopsies excluded decompensated cirrhosis. We do not have further data to make comparisons with these specimens.

11. The data looks normally distributed because it is placed on a log scale to emphasize the nature of variability between individuals. The groups failed D'Aogstino-Pearson testing prior to Kruskal-Wallis testing. We agree with the alternative explanation that you suggest and have added additional emphasis on this. PAS-D does correlate poorly in early fibrosis because there are people with high and low PAS-D who have early fibrosis. However, the high accumulators are of interest to identify with our methods because we suspect that over time they have risk of worsening fibrosis. Our forthcoming longitudinal studies will help clarify this.

12. This is an appropriate criticism and we have edited our discussion to reflect these comments.

13. We analyzed slides to find people at the upper aspects of AAT accumulation regardless of fibrosis stage. Figure 1E is how we identified this group. We then use these groupings throughout the rest of the paper to make further comparisons with people who have high or low accumulation.

14. We used our tools to estimate polymer area in the whole population minus patients with limited tissues or tissue that wouldn't be enough for a pathologist to make an appropriate reading. Figure 3 shows this point. Total IHC does not have a relationship with fibrosis but polymer does.

Minor Comments:

We have made the minor changes as requested.

In this manuscipt we present open source tools developed out of a large cohort of primary biopsy specimens from a rare disease population. We believe that they offer insight into the heterogeneity of disease and cellular damage in early liver fibrosis. We are grateful for the opportunity to share our work with the scientific community through PLOS ONE. We appreciate our reviewers thoughful questions and suggested revisions and have addressed them as above. Please do not hesitate to contact us with any further questions.

Kindest regards,

George Marek MD-PhD
Department of Medicine
Internal Medicine Resident Physician
george.marek@medicine.ufl.edu

Virginia Clark, MD, MS
Associate Professor of Medicine
Division of Gastroenterology, Hepatolgy, and Nutrition
