# Supplementary material for: Quantitative measurement of the histological features of alpha-1 antitrypsin deficiency-associated liver disease in biopsy specimens
Source: PLoS One. 2021 Aug 16;16(8):e0256117. doi: 10.1371/journal.pone.0256117 (PMC8366994; doi:10.1371/journal.pone.0256117)
Supplement: Attachment — Submitted filename: Response to Reviewers.doc [file pone.0256117.s006.doc]

**Department of Medicine**	Alpha 1 Research Program

Division of Pulmonary Critical Care and Sleep Medicine	PO Box 100225

1600 SW Archer Rd, M-333

Gainesville, FL 32611-0225

Dear Dr. Strnad and Review Committee,

Thank you for reviewing our work and offering us the opportunity to incorporate the comments and revisions requested by the review committee. Our paper entitled *Quantitative measurement of the histological features of alpha-1 antitrypsin deficiency-associated liver disease in biopsy specimens* uses whole slide imaging to study baseline histologic data from a large longitudinal cohort of people who have PiZZ alpha-1 antitrypsin deficiency. We demonstrate and provide new tools which can be used to estimate severity of alpha 1 antitrypsin accumulation in standard pathology specimens. We have addressed each reviewers major and minor revisions as below. We have also made editorial revisions as noted.

**Editorial Revisions**

**Reviewer #1**

Reviewer #1 did not have further requests for revision.

**Reviewer #2**

Reviewer #2 had several major revisions and comments.

Reviewer #2: 
*1) The comparisons between fibrosis stage and IHC measurements are not statistically significant. Therefore, how can you say that both polymer and inclusions are both associated with fibrosis?
These sentences should be removed from the abstract and from the discussion (Lines 104, 443).

2) Subgroup Kruskal-Wallis testing with Dunn’s multiple comparisons should not be done (Fig 2) unless the primary analysis is significant. Therefore, the “trend” toward association between polymer and fibrosis can be mentioned. More importantly, you can discuss the reason why you were not able to prove this association (unmeasurable biopsies that limited sample number, etc).*

We performed Dunn’s multiple comparisons test after the primary ANOVA showed differences in the median PAS-D accumulation (94) samples including cirrhosis samples and Polymer IHC measurements (84 samples). Both of these analysis showed significant differences and Post-Hoc analysis was performed at this point. I have attached a charts for these below, and the results are described in the manuscript, I added additional clarification of this in our results section. An important point that we described is that when you stain a biopsy with total AAT immunohistochemistry such that polymeric and non-polymeric AAT is stained and then measured, this is not statistically significant.

**PAS-D Globule Measurements (Figure 2)**

| Table Analyzed | PAS-D Area |
| --- | --- |
|  |  |
| Kruskal-Wallis test |  |
| P value | <0.0001 |
| Exact or approximate P value? | Approximate |
| P value summary | **** |
| Do the medians vary signif. (P < 0.05)? | Yes |
| Number of groups | 5 |
| Kruskal-Wallis statistic | 27.64 |
|  |  |
| Data summary |  |
| Number of treatments (columns) | 5 |
| Number of values (total) | 94 |

**Polymeric AAT Immunohistochemistry (Figure 3)**

| Table Analyzed | 2C1 Area |
| --- | --- |
|  |  |
| Kruskal-Wallis test |  |
| P value | 0.0002 |
| Exact or approximate P value? | Approximate |
| P value summary | *** |
| Do the medians vary signif. (P < 0.05)? | Yes |
| Number of groups | 4 |
| Kruskal-Wallis statistic | 19.29 |
|  |  |
| Data summary |  |
| Number of treatments (columns) | 4 |
| Number of values (total) | 84 |

3) There is an opportunity, even with small patient numbers to create a table of results that might make your trend lines more believable. For instance, if your message is that there is a subset of PAD-D heavy accumulators that have more fibrosis, then define a threshold at which you make your division and show a table of age, race, sex, BMI, PAS-D scores, IHC scores, and fibrosis scoring with statistics comparing the 2 columns of data. If there are other things beside PAS-D stains and fibrosis that are different, then a multivariable analysis is indicated.

This is a really good point and has been a big part of our research with this cohort already. We answered some of these questions when we performed multivariate analysis of AAT Accumulation in Clark et al. 2019 where we defined the clinical changes which are associated with higher accumulators on a qualitative scoring basis by our pathologists. This work was born out of that understanding. In figure 1C we compare these qualitative scores with our quantiative data. The “threshold” you speak of is something that is yet to be defined. The closest we are able to define in this group are outliers based on the statistical distribution of the data but it is unknown whether or not this will have lonigtudinal relevance. Our plan is to define this threshhold based on receiver-operator analysis on progression data as the cohort finishes follow-up such that we know who has developed worstening fibrosis and use these baseline data to determine a clinically relevant amount of accumulation that has sensitivity and specificity to predicting progression.

Minor
1) Fixed
2) Fixed
3) Fixed

Kindest regards,

Virginia Clark, MD, MS

Associate Professor of Medicine

Division of Gastroenterology, Hepatolgy, and Nutrition

George Marek MD-PhD

Department of Medicine

Internal Medicine Resident Physician

george.marek@medicine.ufl.edu
